# Cell Type-Specific p38δ Targeting Reveals a Context-, Stage-, and Sex-Dependent Regulation of Skin Carcinogenesis

**DOI:** 10.3390/ijms20071532

**Published:** 2019-03-27

**Authors:** Alexi Kiss, Aaron C. Koppel, Emily Murphy, Maxwell Sall, Meral Barlas, Grace Kissling, Tatiana Efimova

**Affiliations:** 1 Department of Anatomy & Cell Biology, The George Washington University School of Medicine and Health Sciences, 2300 I Street NW, Ross Hall 550, Washington, DC 20037, USA; kiss@gwu.edu (A.K.); akoppel@gwu.edu (A.C.K.); emurphy@mfa.gwu.edu (E.M.); maxsall@gwmail.gwu.edu (M.S.); meralbarlas@gwmail.gwu.edu (M.B.); 2 The George Washington Cancer Center, 800 22nd Street NW, Science and Engineering Hall 8160, Washington, DC 20052, USA; 3 Department of Dermatology, The George Washington University School of Medicine and Health Sciences, 2150 Pennsylvania Ave NW, Suite 2B-430, Washington, DC 20037, USA; 4 Georgetown University School of Medicine, 3900 Reservoir Rd NW, Washington, DC 20007, USA; 5 Biostatistics and Computational Biology Branch, National Institute of Environmental Health Sciences, Research Triangle Park, NC 27709, USA; GKissling@msn.com

**Keywords:** p38δ/MAPK13, skin carcinogenesis, conditional knockout mice, keratinocytes, myeloid cells

## Abstract

Activation and/or upregulated expression of p38δ are demonstrated in human skin malignancies including cutaneous squamous cell carcinoma, suggesting a role for p38δ in skin carcinogenesis. We previously reported that mice with germline deletion of the p38δ gene are significantly protected from chemical skin carcinogenesis. Here, we investigated the effects of cell-selective targeted ablation of p38δ in keratinocytes and in immune (myeloid) cells on skin tumor development in a two-stage 7,12-dimethylbenz(*a*)anthracene (DMBA)/12-*O*-tetradecanoylphorbol-13-acetate (TPA) chemical mouse skin carcinogenesis model. Conditional keratinocyte-specific p38δ ablation (p38δ-cKO^∆K^) did not influence the latency, incidence, or multiplicity of chemically-induced skin tumors, but led to increased tumor volume in females during the TPA promotion stage, and reduced malignant progression in males and females relative to their wild-type counterparts. In contrast, conditional myeloid cell-specific p38δ deletion (p38δ-cKO^∆M^) inhibited DMBA/TPA-induced skin tumorigenesis in male but not female mice. Thus, tumor onset was delayed, and tumor incidence, multiplicity, and volume were reduced in p38δ-cKO^∆M^ males compared with control wild-type males. Moreover, the percentage of male mice with malignant tumors was decreased in the p38δ-cKO^∆M^ group relative to their wild-type counterparts. Collectively, these results reveal that cell-specific p38δ targeting modifies susceptibility to chemical skin carcinogenesis in a context-, stage-, and sex-specific manner.

## 1. Introduction

Cutaneous squamous cell carcinoma (CSCC) is the second most common type of skin cancer, with an estimated 700,000 or more patients diagnosed annually in the United States [1]. The incidence of CSCC is rising, and the total number of deaths from CSCC is estimated to be similar to that from melanoma [1]. However, our understanding of the mechanisms that contribute to the development and progression of CSCC is limited [2]. Activating mutations in *RAS* genes are widespread in human cancers, including CSCCs [2]. Mitogen-activated protein kinase (MAPK) pathways are among the well-established RAS downstream effector pathways [3]. The MAPK superfamily consists of highly conserved subfamilies of serine/threonine kinases, including extracellular signal-regulated protein kinases (ERK1 and ERK2), stress-responsive c-Jun NH_2_-terminal kinases (JNK1, JNK2, and JNK3), and p38 MAPKs (p38α, p38β, p38γ, and p38δ) [4,5,6,7]. The MAPK signaling pathways control diverse cellular processes in response to a variety of extracellular stimuli. The p38 MAPK family members are activated by environmental stresses, inflammatory cytokines, and growth factors to modulate key cellular processes, including proliferation, differentiation, survival, senescence, migration, and inflammation, in cell type- and context-dependent manners [6,7]. Given that dysregulation of these important processes contributes to tumorigenesis, p38 MAPK signaling is suggested to play a role in cancer development in humans and mice [6,7,8]. However, the in vivo functional contributions of individual p38 MAPKs to tumorigenesis remain to be fully elucidated. 

The p38δ isoform is abundantly expressed in cutaneous epithelia, and is required for appropriate cell proliferation and differentiation in human keratinocyte monolayer and organotypic culture models [9,10]. However, p38δ knockout mice maintain normal skin phenotype [11], likely because of the compensatory functions of the remaining p38 MAPK family members. Notably, upregulated p38δ expression was detected in invasive human CSCC [12], and in several other cancers, including cholangiocarcinoma [13], as well as uterine, ovarian, breast, stomach, colon, and kidney cancers, relative to adjacent normal tissues [14,15]. Moreover, activation of p38δ has been observed in human head and neck SCC [16], suggesting a tumor-promoting function for p38δ in epithelial cancer. Consistent with this notion, significant protective effects of p38δ gene ablation have been demonstrated in several in vivo models of epithelial carcinogenesis [11,17,18]. 

Our laboratory previously reported that mice with systemic (germline) deletion of p38δ were resistant to chemically-induced skin tumorigenesis and to oncogenic K-ras-driven lung tumorigenesis, indicating that p38δ promotes tumor development in vivo [11]. The essential role for p38δ in DMBA/TPA-induced skin tumorigenesis was subsequently confirmed by Zur et al. [17]. We also reported that p38δ gene ablation inhibited the growth of squamous tumors generated from oncogenic v-ras^HA^-transformed keratinocytes following orthotopic grafting onto nude mice by inducing transcriptional changes linked to tumor suppression [18]. These findings suggest that keratinocyte p38δ contributes to oncogenic v-ras^HA^-induced tumorigenesis in a cell-autonomous manner. Furthermore, systemic p38δ loss heightened the initial inflammatory response in pre-neoplastic murine skin following a short-term DMBA/TPA challenge [18]. The correlation between an enhanced acute inflammatory response and significant resistance to DMBA/TPA-induced skin tumor development, reported in several genetically engineered mouse models [19,20,21,22,23,24,25], underscores the critical anti-tumor role of immune/inflammatory factors in the tumor microenvironment. In addition, mice with systemic deletion of both p38δ and p38γ were protected from DMBA/TPA-induced skin tumor development and colitis-associated colon tumorigenesis [17,26]. Systemic p38δ loss was also reported to delay tumor growth, and reduce the number of lung metastases in a murine breast cancer model, suggesting that p38δ promotes breast tumor progression and metastasis [15].

p38δ is expressed not only in epithelial cells, but also in immune, endothelial, and mesenchymal cells; reciprocal communications between these cells and incipient tumor cells have been shown to regulate tumor development and progression. Therefore, the functional involvement of non-epithelial cell-derived p38δ in skin tumorigenesis cannot be excluded. Notably, hematopoietic cell p38γ and p38δ were shown to be the main contributors to colitis-associated tumor initiation in a colorectal cancer mouse model [26]. 

In the present study, we utilized conditional p38δ knockout mice to investigate skin tumor development in response to a two-stage DMBA/TPA chemical skin carcinogenesis protocol. In these mutant mice, genetic ablation of p38δ expression was targeted to keratinocytes (p38δ-cKO^∆K^) or immune (myeloid) cells (p38δ-cKO^∆M^). Cell type-specific loss of p38δ revealed stage- and sex-dependent effects of p38δ inhibition on skin carcinogenesis in vivo, suggesting differential mechanisms of epithelial and myeloid cell p38δ in the regulation of skin tumor development. 

## 2. Results

### 2.1. Mice Lacking Keratinocyte p38δ Exhibit a Normal Skin Phenotype

**Figure 1:**
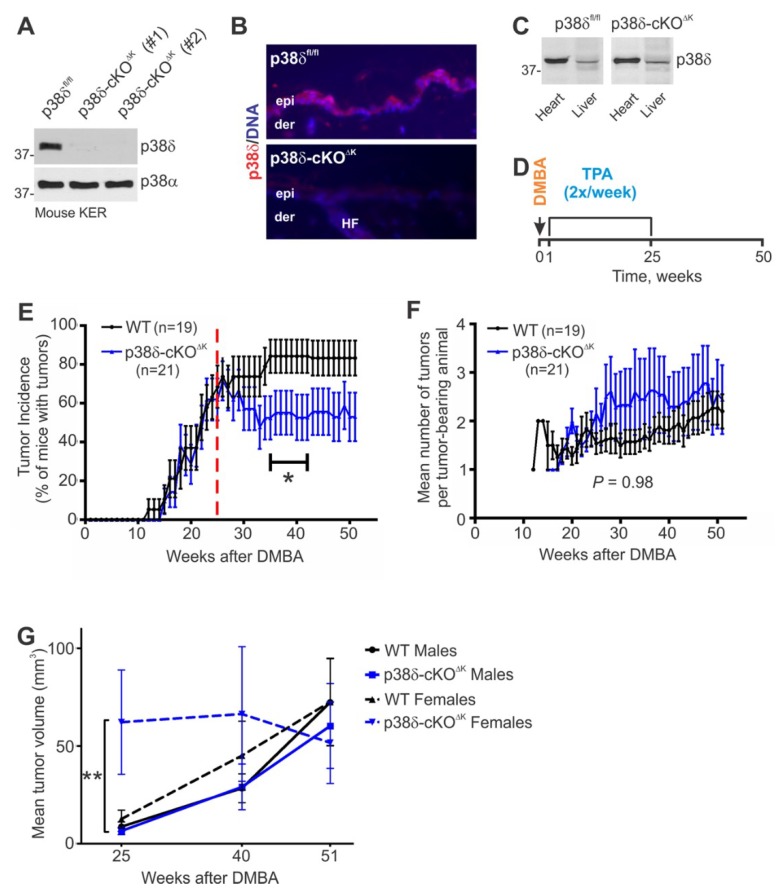
The effects of keratinocyte p38δ deficiency on skin tumor development. (**A**) Immunoblot analysis of p38δ and p38α expression in total cell extracts prepared from cultured mouse keratinocytes isolated from newborn WT control (p38δ^fl/fl^) and mutant (p38δ-cKO^ΔK^, #1 and #2) littermates. (**B**) Immunofluorescence analysis of p38δ expression in p38δ^fl/fl^ and p38δ-cKO^ΔK^ adult mouse epidermis. Skin sections from eight-week-old mice were stained with antibody against p38δ. Nuclei were counterstained with Hoechst. Epi, epidermis; der, dermis; HF, hair follicle. Representative tissue fields. Magnification, 20×. (**C**) Immunoblot analysis of p38δ expression in heart and lung tissue lysates from p38δ^fl/fl^ and p38δ-cKO^ΔK^ littermate mice. (**D**) Schematic depicting the DMBA/TPA treatment regimen. (**E**) Tumor incidence (mean ± SE). p38δ-cKO^ΔK^ mice (K14-Cre^+/−^; p38δ^flox/flox^) were compared with their WT control littermates (p38δ^flox/flox^ and p38δ^flox/+^) by their responses to the two-stage DMBA/TPA chemical carcinogenesis regimen shown in (**D**). Male and female mice of both genotypes were used in the study; sex ratios did not differ significantly between the WT and p38δ-cKO^ΔK^ mice: *p* = 0.5365; Chi-square test. The dashed line marks the week of the last TPA application (Week 25 post-DMBA). * Tumor incidence of p38δ-cKO^ΔK^ mice differed significantly from that of WT mice between 35 and 42 weeks post-DMBA: *p* < 0.05; Fisher’s exact test. (**F**) Tumor multiplicity (mean ± SE). (**G**) Tumor volume (mean ± SE). Individual tumor volumes were calculated based on the formula: Volume, (mm^3^) = *π* × (radius)^2^ × height; individual tumors were treated as the units of analysis (see also Table S2d). ** *p* = 0.003; one-sided Mann–Whitney test.

To determine if the loss of keratinocyte-intrinsic p38δ influences chemically-induced skin tumor development, we generated mice with epidermal keratinocyte-specific deletion of p38δ (Ker14-Cre^+/−^; p38δ^flox/flox^:p38δ-cKO^∆K^). We observed efficient p38δ ablation in keratinocytes, while the levels of p38δ expression in heart and liver remained unchanged, indicating that the p38δ ablation was keratinocyte-specific (Figure 1A–C). In contrast, p38α protein was similarly expressed in WT and mutant keratinocytes (Figure 1A). Consistent with the observed normal skin phenotype in mice with systemic (germline) p38δ gene ablation [11], the p38δ-cKO^∆K^ epidermis lacked discernable abnormalities (Figure 1B, and data not shown), indicating that p38δ expression in epidermal keratinocytes is not essential for skin development, postnatal growth, and homeostasis. To elucidate the role of keratinocyte p38δ during skin tumor development, we subjected groups of WT and p38δ-cKO^∆K^ male and female mice to the DMBA/TPA skin carcinogenesis regimen detailed in the Section 4 and outlined in Figure 1D.

### 2.2. Keratinocyte-Specific Loss of p38δ Leads to Reduced Tumor Incidence during the Malignant Progression Stage of the DMBA/TPA Regimen and to Increased Tumor Growth in Females during the TPA Promotion Stage

As shown in Table S1, tumor latency (time to first tumor) did not differ significantly between WT and p38δ-cKO^∆K^ genotypes. Although loss of p38δ in keratinocytes did not affect tumor incidence during the TPA promotion stage (Weeks 1–25 post-initiation with DMBA), the incidence of tumors in p38δ-cKO^∆K^ mice was significantly reduced during the malignant progression stage of the DMBA/TPA regimen (Figure 1E). However, tumor multiplicity was not significantly affected by keratinocyte p38δ loss over the course of the experiment (Figure 1F). Analysis by sex revealed that mean tumor volume was significantly increased over 10-fold in p38δ-cKO^∆K^ female mice compared to WT female mice at Weeks 7 and 8 after individual tumor detection during the TPA promotion stage (Table S2a). Analysis of tumor volumes in males and females separately and together at Weeks 25, 40, and 51 post-DMBA revealed no significant differences between the WT and p38δ-cKO^∆K^ genotypes (Table S2b,c). However, at Week 25 post-DMBA (end of the TPA promotion stage), the mean tumor volume of mutant female mice was significantly larger than that of mutant male mice (Figure 1G and Table S2d). Remarkably, the rapid tumor growth seen in p38δ-cKO^∆K^ female mice ceased upon termination of inflammation-inducing TPA treatments (Figure 1G), indicating a critical role of TPA-induced pro-inflammatory signaling in promoting tumor growth in female mice with keratinocyte p38δ loss. 

### 2.3. Keratinocyte p38δ Deletion Does Not Affect Induction of Inflammatory Cytokines in Response to a Short-Term DMBA/TPA Regimen in Mouse Skin

**Figure 2:**
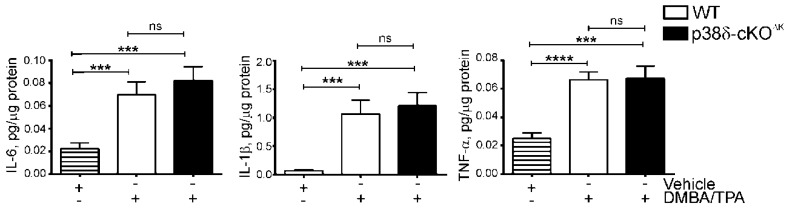
Keratinocyte-specific p38δ deletion does not affect induction of inflammatory cytokines in response to a short-term DMBA/TPA regimen. Sets of WT and p38δ-cKO^ΔK^ littermates of both sexes were subjected to a topical short-term DMBA/TPA treatment as detailed in the Materials and Methods or treated with acetone vehicle. Total skin lysates were isolated from full-thickness dorsal skin of mice (acetone vehicle control: *n* = 8; DMBA/TPA: *n* = 20 (WT) and *n* = 17 (p38δ-cKO^ΔK^)) 2 h after the final TPA treatment, and the levels of protein expression of the indicated cytokines were analyzed using ELISA. Results are shown as mean ± SE. *** *p* < 0.001, **** *p* < 0.0001; ns, not significant; vehicle treated group included mice of both genotypes.

To examine the effect of keratinocyte-specific p38δ deletion on the early DMBA/TPA-induced inflammatory response in pre-neoplastic mouse skin, we used ELISA to assess the levels of inflammatory cytokines in the skin of WT and p38δ-cKO^∆K^ mice after a short-term DMBA/TPA regimen. Comparing combined or separate male and female cohorts of WT and mutant mice, we observed that keratinocyte p38δ loss did not significantly influence the DMBA/TPA-stimulated induction of TNFα, IL-1β, or IL-6 proteins (Figure 2 and Figure S1). Thus, these results show that ablation of p38δ in keratinocytes does not affect inflammatory cytokine production in the pre-neoplastic skin of mice subjected to a short-term DMBA/TPA challenge.

### 2.4. p38δ-cKO^∆K^ Mice Exhibit a Reduced Incidence of Malignant Tumors

As shown in Table S3a, the incidence of malignant tumors (SCCs and keratoacanthomas, KAs) was significantly reduced in mutant p38δ-cKO^∆K^ mice compared to their WT counterparts at the end of the carcinogenesis experiment (Week 51 post-DMBA). In addition, total and malignant tumor multiplicities were significantly reduced in p38δ-cKO^∆K^ female mice compared to WT females, and benign tumor multiplicity was significantly lower in mutant female mice than mutant male mice (Table S3b,c). These findings show that malignant progression was impaired in p38δ-cKO^∆K^ mice relative to WT control mice.

Collectively, our results using mice with conditional keratinocyte-specific ablation of p38δ showed that keratinocyte-intrinsic p38δ function was dispensable for the initiation and promotion stages of DMBA/TPA skin tumor development, but was essential for the malignant progression of DMBA/TPA-induced skin tumors. In addition, keratinocyte-specific p38δ loss modified susceptibility to chemical skin tumorigenesis in a sex-specific manner, resulting in increased tumor growth only in female mice during the TPA promotion phase.

### 2.5. Loss of p38δ in Myeloid Cells Impairs Development of DMBA/TPA-Induced Skin Tumors in Male Mice 

Myeloid cells, including macrophages, neutrophils, monocytes, dendritic cells, and granulocytes, are recruited to the tumor microenvironment to regulate tumor growth and progression (reviewed in [27,28,29]). We theorized that myeloid cell-intrinsic p38δ [30] may regulate skin tumor development. To explore the impact of myeloid cell-intrinsic p38δ loss on DMBA/TPA-stimulated skin tumorigenesis, we generated mice with conditional myeloid cell-specific p38δ deletion (LysM-Cre^+/−^; p38δ^flox/flox^:p38δ-cKO^ΔM^). The efficiency of the deletion of p38δ in myeloid cells using this approach was previously confirmed [31]. Consistent with the lack of overt skin abnormalities in mice with systemic p38δ loss [11], the epidermis of p38δ-cKO^ΔM^ newborn and adult mice was histologically indistinguishable from that of WT control littermates (data not shown). We then subjected cohorts of WT and p38δ-cKO^ΔM^ male and female mice to the DMBA/TPA skin carcinogenesis regimen described in the Materials and Methods and outlined in Figure 1D.

**Figure 3:**
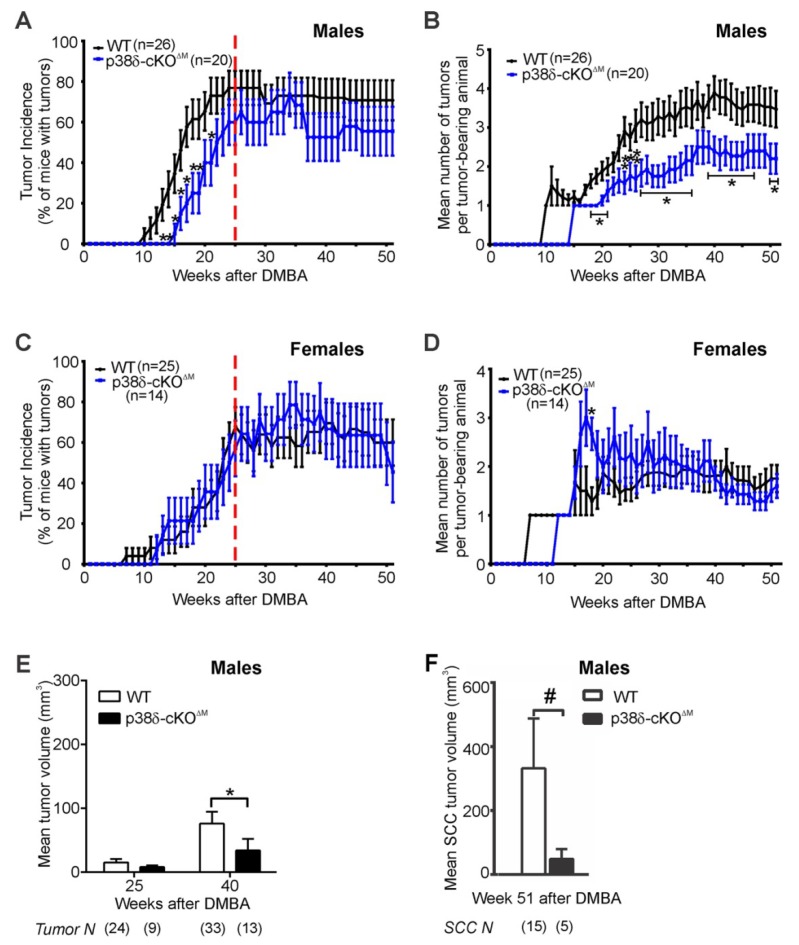
The effects of myeloid cell-specific p38δ deficiency on skin tumor development. p38δ-cKO^ΔM^ male or female mice (LysM-Cre^+/−^; p38δ^flox/flox^) were compared with their WT control counterparts (p38δ^flox/flox^ and p38δ^flox/+^) by their responses to the two-stage DMBA/TPA chemical carcinogenesis regimen. (**A**,**C**) Tumor incidence (mean ± SE). The dashed line marks the week of the last TPA application (Week 25 post-DMBA). * *p* < 0.05; Fisher’s exact test. (**B**,**D**) Tumor multiplicity (mean ± SE). * *p* < 0.05; ** *p* < 0.01; Fisher’s exact test. (**E**,**F**) Tumor volume (mean ± SE). Individual tumor volumes were calculated based on the formula: Volume, (mm^3^) = π × (radius)^2^ × height; * *p* < 0.05; # approaching significance at *p* = 0.0581; one-sided Mann–Whitney test was used to compare tumor volumes between genotypes at the indicated time points, using the individual tumors as the units of analysis. The numbers of tumors per each group are shown in parenthesis.

**Figure 4:**
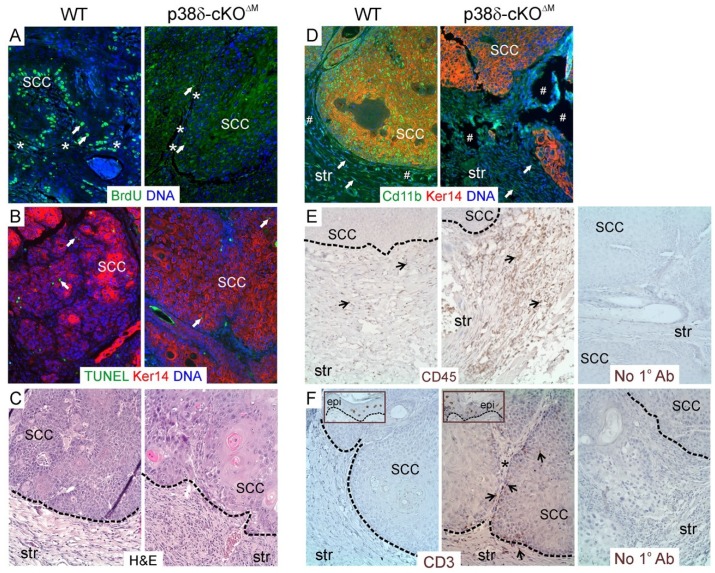
SCC tumors from myeloid p38δ-deficient male mice exhibit reduced tumor cell proliferation, similar levels of apoptosis, and increased leukocyte recruitment into the peritumoral stroma compared with SCC tumors from WT male mice. Representative sections of WT and p38δ-cKO^ΔM^ SCC tumors, collected after the completion of the carcinogenesis experiment, are shown; *n* = 3 tumor samples per genotype were examined. (**A**) Immunofluorescence staining for BrdU (green) showing markedly reduced numbers of BrdU^+^ nuclei in p38δ-cKO^ΔM^ SCC tumors compared with WT SCC tumors. White arrows point to representative BrdU-positive nuclei. Asterisks mark regions of intratumoral stroma. (**B**) Immunofluorescence staining for TUNEL and Keratin 14 (Ker14). White arrows point to representative TUNEL^+^ apoptotic nuclei (green). Ker14 positivity (red) marks epithelial compartment of SCCs. (**C**) H&E staining shows a higher density of inflammatory cells in the peritumoral stroma of p38δ-cKO^ΔM^ SCC tumors compared with WT tumors. (**D**) Immunofluorescence staining for CD11b (green) and Ker14 (red). White arrows point to representative CD11b^+^ myeloid cells in the peritumoral stroma of SCCs. Note the higher density of CD11b^+^ cells in the stroma of p38δ-cKO^ΔM^ SCC tumors. # marks regions of poor tissue preservation. (**E**) Immunohistochemical staining for CD45 pan-leukocyte marker shows increased leukocyte infiltration into the peritumoral stroma of p38δ-cKO^ΔM^ SCC tumors compared with WT SCC tumors. Black arrows point to representative CD45^+^ cells. (**F**) Immunohistochemical staining for CD3 (T cells, brown). Black arrows point to representative CD3^+^ cells. CD3^+^ cells are seen in the parenchyma as well as in the intratumoral (asterisk) and peritumoral stroma of p38δ-cKO^ΔM^ SCC tumors, but not in WT SCC tumors. Insets show CD3^+^ dendritic epidermal T cells in epidermis of WT and p38δ-cKO^ΔM^ skin adjacent to the corresponding SCC tumors, providing an intrinsic positive control for CD3 staining. In (**A**,**B**,**D**), nuclei were counterstained with Hoechst dye (DNA). In (**C**,**E**,**F**), dashed line demarcates tumor-stromal boundaries. In (**E**,**D**), no primary antibody panels (No 1^o^ Ab) are included as negative controls. SCC, squamous cell carcinoma; str, stroma; epi, epidermis; Magnification, 20×.

Our data showed that tumor latency was significantly increased in male p38δ-cKO^ΔM^ mice compared to WT males (Table 1), and tumor incidence was significantly reduced in mutant p38δ-cKO^ΔM^ males relative to their WT counterparts during the TPA promotion stage (Figure 3A). Tumor multiplicity was significantly lower in mutant male mice compared to WT males throughout the course of the carcinogenesis experiment (Figure 3B). In contrast, tumor latency, incidence, and multiplicity did not differ significantly between the WT and p38δ-cKO^ΔM^ genotypes in female mice (Table 1 and Figure 3C,D). In addition, mean tumor volume was significantly reduced in p38δ-cKO^ΔM^ males compared to WT males at Week 41 post-DMBA (Figure 3E). In contrast, measurement of tumor volume at Weeks 25, 41, and 51 post-DMBA revealed no significant differences between the WT and mutant genotypes in female mice (data not shown). Furthermore, at the completion of the DMBA/TPA carcinogenesis protocol (Week 51 post-DMBA), p38δ-cKO^ΔM^ males showed a decreased incidence of malignant tumors relative to WT males (Table S4), and the mean volume of malignant SCCs was reduced in p38δ-cKO^ΔM^ males compared to WT males (Figure 3F). SCCs collected from p38δ-cKO^ΔM^ male mice at the end of the carcinogenesis study exhibited dramatically reduced proliferation relative to SCCs isolated from WT males, as determined by BrdU staining (Figure 4A). In contrast, the levels of apoptosis, as assessed by TUNEL staining, were not affected by myeloid cell-specific p38δ deletion—immunofluorescence analysis showed very few TUNEL-positive apoptotic cells in the epithelial compartments of SCCs from WT and mutant male mice (Figure 4B). We also observed an increased accumulation of macrophages and granulocytes (CD11b^+^), leukocytes (CD45^+^), and T cells (CD3^+^) in the peritumoral stroma of SCCs from p38δ-cKO^ΔM^ male mice compared to WT male mice (Figure 4C–F). In addition, we also detected CD3^+^ cells in the parenchyma as well as in the intratumoral stroma of p38δ-cKO^∆M^ SCC tumors from male mice, but not in SCC tumors from their WT counterparts. These results suggest that myeloid p38δ loss limits growth of the DMBA/TPA-induced SCC tumors in male mice, likely by promoting a tumor-suppressive immune microenvironment.

Taken together, our data show that myeloid cell-specific p38δ loss modifies susceptibility to chemical skin carcinogenesis in a sex-specific manner and suggest that myeloid cell p38δ is essential for DMBA/TPA-induced skin tumor development in male mice, but not in female mice. 

## 3. Discussion

Using conditional knockout mice lacking p38δ in epidermal keratinocytes or myeloid cells, we uncovered important context-, stage-, and sex-dependent in vivo functional roles for p38δ in the promotion of DMBA/TPA chemical skin carcinogenesis. 

We previously found that systemic (germline) genetic deletion of p38δ inhibited DMBA/TPA-induced skin tumorigenesis via attenuation of the proliferative ERK1/2-AP1 pathway and suppressed K-Ras-driven lung tumorigenesis, indicating a tumor-promoting function for p38δ in skin and lung tissue in vivo [11]. Others have confirmed a critical role of p38δ in DMBA/TPA-induced skin tumorigenesis [17]. We also previously showed that the loss of p38δ was associated with hyper-activation of p38α and enhanced inflammation in response to stimuli such as short-term DMBA/TPA skin treatment and TNFα treatment of cultured mouse keratinocytes [18].

Our present studies revealed that, unlike systemic p38δ ablation, conditional ablation of p38δ in epidermal keratinocytes did not protect mice from skin tumor development during the initial stages of the DMBA/TPA regimen, suggesting that keratinocyte-intrinsic p38δ function is not necessary for the initiation and promotion stages of DMBA/TPA skin tumor development. In contrast, during the malignant progression stage of the DMBA/TPA regimen, p38δ-cKO^ΔK^ mice exhibited significantly reduced overall and malignant tumor incidences compared to WT mice at the end of the carcinogenesis experiment (51 weeks post-initiation with DMBA), indicating that keratinocyte-intrinsic p38δ functionally contributes to the malignant progression of DMBA/TPA-induced skin tumors. 

Our studies further showed that keratinocyte-specific targeting of p38δ modifies susceptibility to DMBA/TPA-induced skin tumor development in a sex-specific manner, resulting in significantly increased tumor growth in only female mice during the TPA promotion stage. This phenomenon likely involves a p38α/Estrogen Receptor (ER)-dependent signaling mechanism, given that the p38α isoform is hyper-activated in initiated keratinocytes and incipient tumor cells in the absence of p38δ [18], and that p38α has been shown to mediate ER transcriptional activation and signaling to promote cancer cell proliferation and survival [32,33,34,35]. For instance, p38α/ER-stimulation of p38δ-proficient endothelial and/or immune cells could potentially promote tumor proliferation and/or angiogenesis during the TPA promotion stage in female p38δ-cKO^ΔK^ mice, in which p38α is hyper-activated upon keratinocyte p38δ loss in response to TPA-induced pro-inflammatory stimulation [18]. This hypothesis needs to be tested in future studies. Importantly, despite the initial rapid dynamics of tumor growth in mutant female mice, p38δ-cKO^ΔK^ female animals showed significantly lower total and malignant tumor multiplicities than their WT counterparts at the end of the carcinogenesis experiment (Table S3), underscoring the essential role of keratinocyte p38δ in promoting the malignant progression of chemically-induced tumors in female mice. 

Furthermore, using immune (myeloid) cell-specific p38δ targeting, we demonstrated that male, but not female, mice with myeloid p38δ loss were significantly protected from DMBA/TPA-induced skin tumor development during both the promotion and malignant progression stages of the DMBA/TPA regimen, and exhibited an increased tumor latency, as well as reduced tumor incidence, multiplicity, and volume, compared to WT male littermates. In addition, mutant p38δ-cKO^ΔM^ male mice had a reduced incidence of malignant tumors, suggesting that myeloid cell-intrinsic p38δ is essential for malignant progression of DMBA/TPA-induced skin tumors in males. These findings support the notion that tumor-extrinsic myeloid cell-derived p38δ functionally contributes to chemically-induced skin tumor development in male mice. 

Inflammatory cells are recognized as a key component of the tumor microenvironment [36,37,38]. Specifically, several subsets of innate immune cells of myeloid origin, including monocytes, macrophages, granulocytes/neutrophils, mast cells, and some subgroups of lymphocytes, constitute major components of the leukocyte infiltrate in tumors and are known to dynamically regulate tumor growth and progression [36,37,38]. Myeloid cells in the tumor microenvironment could have tumor-promoting or tumor-destructing properties by adopting either pro-tumorigenic immunosuppressive T_H_2 or anti-tumorigenic immune-stimulatory T_H_1 states, respectively [27,36,37,38]. Further, the hypothesis that T_H_2-driven myeloid cells could be reprogrammed to instead promote anti-tumor immunity has been tested in several tissue-specific cancer models (reviewed in [36]). Increased infiltration of the peritumoral stroma by macrophages and granulocytes (CD11b^+^), leukocytes (CD45^+^), and T cells (CD3^+^) seen in our data correlated with reduced SCC tumor size and reduced tumor cell proliferation in p38δ-cKO^ΔM^ male mice compared to their WT counterparts (Figure 4C–F). This suggests that myeloid p38δ targeting could contribute to reprogramming of the inflammatory myeloid cell phenotype, ultimately mediating increased anti-tumor immunity and impaired SCC tumor growth in males. Relevantly, we previously reported that the expression of genes linked to the Myeloid Leukocyte Activation gene category was significantly downregulated by p38δ ablation in v-ras^HA^-initiated mouse keratinocytes [18]. Specifically, among the down-regulated genes, listed in the Table S5, are TGFβ receptor II (*TGFBR2*) and Fcγ receptors (*FCGR2B* and *FCGR3*). Given that TGFβ has been implicated in induction of pro-tumorigenic M2 macrophage and N2 neutrophil polarization, while inhibition of TGFβ signaling resulted in switching to an anti-tumorigenic N1 phenotype (reviewed in [39]), and that Fcγ receptor signaling in myeloid cells has been shown to regulate inflammation-associated squamous carcinogenesis via reprogramming myeloid cell phenotypes [40], it is conceivable that myeloid p38δ loss could impact the bioactive state of myeloid cells, in part through regulation of the above mentioned genes. Further studies are needed to determine the precise mechanisms of this regulation.

An unexpected aspect of our findings was uncovering the sex-dependent differences in specific features of the DMBA/TPA chemical skin carcinogenesis process in both p38δ-cKO^ΔK^ and p38δ-cKO^ΔM^ mice, supporting the significance of the sex-specific role p38δ signaling plays in both the tumor parenchyma and myeloid component of the tumor stroma. 

In summary, by focusing on functional outcomes of cell type-specific p38δ genetic targeting in an experimental model of chemically-induced mouse skin carcinogenesis, our findings support that p38δ has essential in vivo context-, stage-, and sex-dependent roles in cutaneous carcinogenesis, and contributes both cell-autonomous and paracrine effects during skin tumor formation. Overall, along with work by other groups, our data suggest that there are potential therapeutic benefits of targeting p38δ for the treatment of cancer. Furthermore, our data provide a rationale to validate an innovative approach of cell-specific targeting of p38δ for the treatment of cancer in a sex-optimized manner. The regulatory mechanisms underlying sex disparities in skin carcinogenesis have yet to be elucidated; our mouse models with cell-specific p38δ ablation will be instrumental in addressing these disparities at the molecular level in an in vivo setting.

## 4. Materials and Methods

### 4.1. Reagents and Antibodies 

DMBA, TPA, bromodeoxyuridine (BrdU), Hoechst 33342, and p38α antibody were purchased from Sigma (St. Louis, MO, USA). For immunofluorescence experiments, we used p38δ antibody obtained from Santa Cruz Biotechnology (Santa Cruz, CA, USA). BrdU antibody was from Chemicon (Temecula, CA, USA). For immunoblot experiments, we used p38δ antibody purchased from the Division of Signal Transduction Therapy (Dundee, UK) [41]. Antibodies against CD45 and CD11b were from Thermo Fisher Scientific (Waltham, MA, USA), and CD3 antibody was from Rockland Immunochemicals (Limerick, PA, USA). Antibody against keratin 14 was obtained from Covance Research Products (Berkeley, CA, USA). 

### 4.2. Generation of Mice with Keratinocyte- and Myeloid Cell-Specific Ablation of p38δ

All animal studies were approved by the Washington University School of Medicine (WUSM) Animal Studies Committee (Animal Welfare Assurance # A-3381-01, approved on 20 July 2012). Mice were housed under pathogen-free conditions and handled in accordance with National Institutes of Health guidelines. Mice with *loxP*-flanked alleles of *Mapk13*, the gene encoding p38δ, (p38δ^flox/flox^), were generously supplied by Professor Romeo Ricci, with permission from Boehringer Ingelheim Pharmaceuticals Inc. (Ridgefield, WA, USA). The p38δ floxed allele was generated by homologous recombination in embryonic stem cells in which the first exon (containing ATG) was flanked by two LoxP sites. The detailed description of the targeting strategy was provided by Sumara et al. [42]. p38δ^flox/flox^ mice were backcrossed to a C57BL/6 genetic background for at least six generations. Mice with keratinocyte- and myeloid cell-specific ablation of p38δ were generated by crossing p38δ^flox/flox^ mice with Keratin 14 (K14)-Cre [43] and Lysozyme M (LysM)-Cre transgenic mice [44], respectively. K14-Cre and LysM-Cre transgenic mice were obtained from The Jackson Laboratory. Genotyping PCR was performed by Mouse Genetics Core at Washington University in St. Louis using genomic mouse tail DNA samples. Conditional mutants p38δ-cKO^ΔK^ (K14-Cre^+/−^; p38δ^flox/flox^) and p38δ-cKO^ΔM^ (LysM-Cre^+/−^; p38δ^flox/flox^) were compared to their wild-type control littermates (p38δ^flox/flox^ and p38δ^flox/+^) by their response to the two-stage DMBA/TPA chemical carcinogenesis regimen.

### 4.3. Chemical Skin Carcinogenesis

Groups of 8-week-old male and female mice were subjected to the DMBA/TPA tumor induction regimen as previously detailed [11]. Briefly, two days after shaving, a single initiating dose of topical DMBA (100 μg in 200 μL acetone per mouse) was applied to the dorsal skin of mice. One week later, tumor promoter TPA (12.5 μg in 200 μL acetone per mouse) was applied to the same site two times per week for 25 weeks. A schematic of the DMBA/TPA treatment regimen is shown in Figure 1D. The onset of tumor formation as well as the number and size of tumors were recorded weekly. Following termination of the TPA treatments, the mice were monitored for the conversion of benign papillomas to SCCs and tumor tissues were harvested for histological verification at Week 51 post-initiation with DMBA. Histopathological evaluation of tumors was carried out by Dr. Suellen Greco in the Division of Comparative Medicine at WUSM.

### 4.4. A Short-Term DMBA/TPA Treatment

A short-term topical DMBA/TPA treatment was carried out as previously detailed [18]. Briefly, DMBA (100 μg in 200 μL acetone per mouse) or acetone vehicle was topically applied to shaved dorsal skin of sets of 12–15-week-old WT and p38δ-cKO^ΔK^ male and female littermates two days after shaving. Starting 5 days post-DMBA or vehicle treatment, mice were treated every other day with four topical applications of TPA (12.5 μg in 200 μL acetone per mouse per each dose) or acetone vehicle. Total skin lysates were isolated from full-thickness dorsal skin 2 h after the final TPA application.

### 4.5. Tissue Protein Isolation, Immunoblot Analysis and ELISA

Full-thickness dorsal skin samples were collected, protein lysates were prepared, protein concentrations of the lysates were determined, and immunoblot analysis was performed as previously detailed [11,18]. IL-1β, IL-6, and TNFα levels in the full-thickness skin lysates were measured using the appropriate R&D Systems DuoSet ELISA Kits according to the manufacturer’s recommendation (R&D Systems, Minneapolis, MN, USA), as previously reported [18]. ELISA reactions from each sample were measured in duplicates. Tissue cytokine levels were determined by normalizing the interpolated cytokine amounts to the total protein concentration of the respective sample and expressed as pg cytokine/µg protein for each skin lysate sample. Normalized skin tissue cytokine levels between experimental animal groups of different genotypes were compared using ANOVA, and pair-wise comparisons were performed using two-tailed, unpaired Student’s *t*-test (PRISM software, GraphPad Software, San Diego, CA, USA) to determine statistically significant differences.

### 4.6. Histology, Immunofluorescence and Immunohistochemistry

Dorsal skin or tumor samples were fixed in 4% paraformaldehyde in PBS, dehydrated in ethanol, embedded in paraffin, and sectioned at 5 μm. For immunofluorescence detection, sections were deparaffinized, rehydrated, and subjected to antigen retrieval by microwaving in 10 mM citrate buffer (pH 6.0). After blocking, sections were stained with primary antibody at 4 °C overnight in a humid chamber, followed by incubation with the appropriate fluorochrome-conjugated secondary antibody for 2 h at room temperature. Nuclei were stained with Hoechst 33342 dye (Sigma, St. Louis, MO, USA). Immunohistochemistry was performed on paraffin-embedded sections subjected to antigen retrieval as described above. After blocking, sections were incubated with primary antibodies at 4 °C overnight in a humid chamber, followed by incubation with biotinylated IgG at RT for 30 min. Detection of the signal was carried out using Vectastain ABC kit (Vector Laboratories, Burlingame, CA, USA) and diaminobenzidine (DAB) substrate kit (Vector Laboratories, Burlingame, CA, USA) according the manufacturer’s instructions. The sections were counterstained with hematoxylin. Microscopy was performed using a fluorescence microscope (Nikon Eclipse E600), Spot RT3 camera and Spot imaging software.

### 4.7. Statistical Methods

Statistical analyses were carried out as previously detailed [18]. Briefly, we used one-sided log-rank tests to compare time to tumor (latency) among genotypes, and one-sided Mann–Whitney tests to compare numbers of tumors per tumor-bearing animal (multiplicity) between genotypes at each week. We also used repeated measures analysis of variance (ANOVA) to compare multiplicity between genotypes. For weekly number of tumor-bearing animals (incidence), we used one-sided Fisher’s exact test.

## Figures and Tables

**Table 1:** Tumor latency (time to first tumor) was significantly delayed in male mice with myeloid cell-specific ablation of p38δ (p38δ-cKO^ΔM^) compared to WT males.

Title	Males and Females		Males		Females	
	WT	p38δ-cKO^ΔM^	WT	p38δ-cKO^ΔM^	WT	p38δ-cKO^ΔM^
Number of animals	51	34	26	20	25	14
Median time to tumor (weeks)	21	23	16.5	22.5	23	23.5
Ratio of median time until tumor	0.913		0.7333		0.9787	
95% confidence interval of ratio of median time until tumor	0.2985–1.528		0.2146–1.252		0.4966–1.461	
Mean time to tumor ± SE	24.4 ± 1.9	28.3 ± 2.4	21.9 ± 2.6	28.8 ± 3.1	26.9 ± 2.6	27.5 ± 3.7
Log-rank test *p*-values (one-sided)	0.0949		0.0291 (*)		0.4371	

* *p*-value < 0.05.

## Supplementary Materials

Supplementary materials can be found at https://www.mdpi.com/1422-0067/20/7/1532/s1.

## Abbreviations

SCC	squamous cell carcinoma
CSCC	cutaneous squamous cell carcinoma
KA	keratoacanthoma
DMBA	7,12-dimethylbenz(*a*)anthracene
TPA	12-*O*-tetradecanoylphorbol-13-acetate
cKO	conditional knockout
ER	estrogen receptor
