# Supplementary material for: Cell Type-Specific p38δ Targeting Reveals a Context-, Stage-, and Sex-Dependent Regulation of Skin Carcinogenesis
Source: Int J Mol Sci. 2019 Mar 27;20(7):1532. doi: 10.3390/ijms20071532 (PMC6479675; doi:10.3390/ijms20071532)
Supplement: Supplementary file 1 [file ijms-20-01532-s001.pdf]

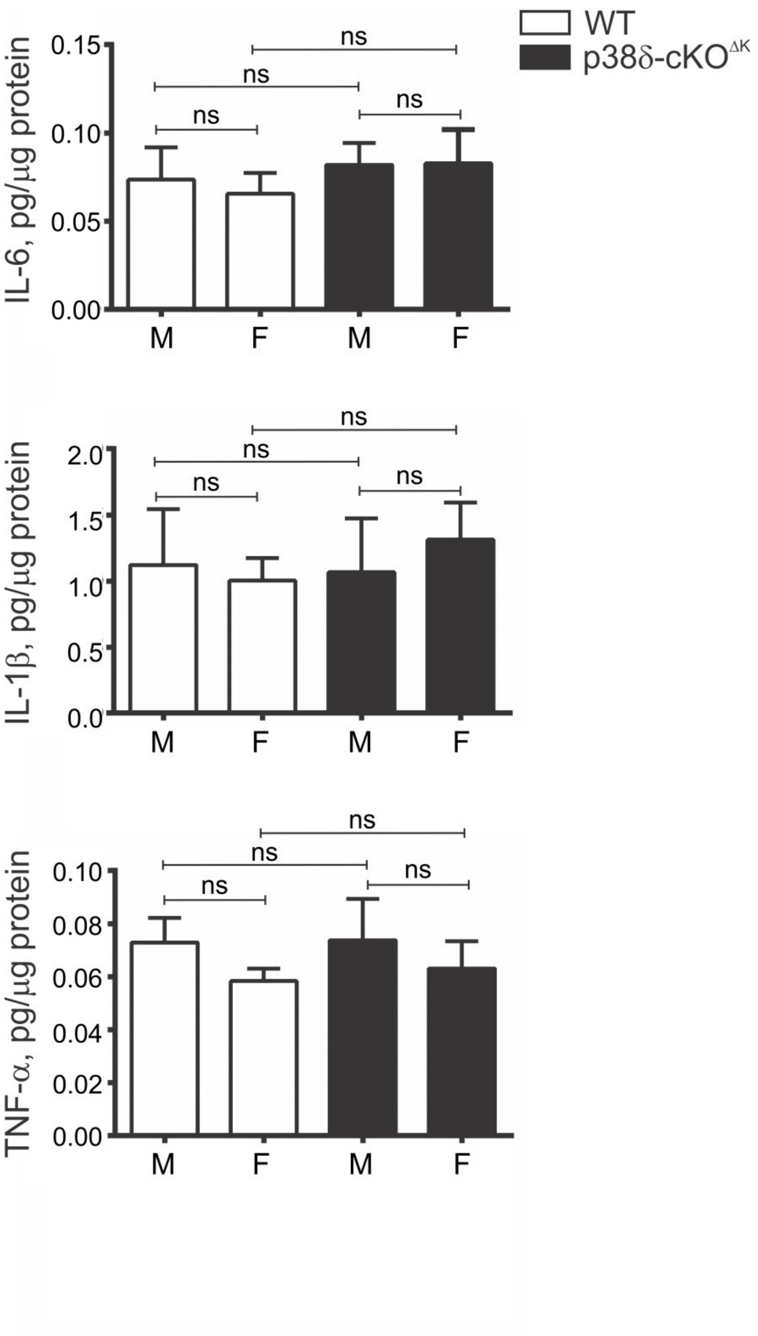

Supplementary Figure S1. Analysis of sex-specific inflammatory cytokine expression in WT and p38δ-cKO$^{\Delta K}$ male (WT males: n = 11, cKO males: n = 7) and female (WT females: n = 9; cKO females: n = 10) mouse skin subjected to a short-term DMBA/TPA regimen as detailed in the Materials and Methods. Total skin lysates were isolated from full-thickness dorsal skin 2 h after the final TPA treatment, and levels of protein expression of the indicated cytokines were analyzed using ELISA. Results are shown as mean ± SE. ns, not significant; M, males; F, females.

Supplementary Table S1

Tumor latency (time to first tumor) did not differ significantly between the WT and p38δ-cKO$^{\Delta K}$ genotypes.

We used one-sided log-rank tests to compare tumor latency between genotypes.

	WT	p38δ-cKO$^{\Delta K}$
**Number of animals**	19	21
**Median time to tumor (weeks)**	22	23
**95% confidence interval for median**	18, 25	18, 25
**Mean time to tumor ± s.e.**	22.8 ± 1.6	23.4 ± 1.5
**Number of tumor-free animals at the end of study (%)**	1	2
**Log-rank p-values**		
**WT vs.**		0.3520

Supplementary Tables S2

Tumor volume comparisons between WT and p38δ-cKO^{ΔK} genotypes.

(In the analyses below, the tumor was treated as the unit of analysis.)

a) The ratios of mean individual tumor volumes in female mice were compared between the WT and p38δ-cKO^{ΔK} genotypes at the indicated week after detection during TPA promotion stage; ns, not significant.

Week after detecting individual tumors in females during TPA promotion	Ratio of mean tumor volumes of genotypes (p38δ-cKO^{ΔK}/ WT)	Significance by Mann-Whitney test (one-sided)	p-value
1	1.2	ns	0.1086
2	1.3	ns	0.2791
3	1.0	ns	0.4024
4	2.4	ns	0.0955
5	0.8	ns	0.3141
6	1.1	ns	0.2051
7	**10.6**	**	**0.0079**
8	**10.9**	*	**0.0357**
9	13.0	ns	0.0667

b) Tumor Volume, Mean ± s.e. There were no significant differences between the WT and p38δ-cKO^{ΔK} genotypes for any group at any time point.

	WT			p38δ-cKO^{ΔK}		
	M	F	M&F	M	F	M&F
25 weeks	8.7 ± 2.4 (n = 15)	12.6 ± 4.6 (n = 4)	9.5 ± 2.1 (n = 19)	6.5 ± 1.8 (n = 20)	62.2 ± 26.6 (n = 9)	23.8 ± 9.4 (n = 29)
40 weeks	28.4 ± 7.4 (n = 16)	45.1 ± 17.7 (n = 12)	35.6 ± 8.6 (n = 28)	29.1 ± 11.7 (n = 14)	66.4 ± 34.4 (n = 7)	41.5 ± 13.9 (n = 21)
51 weeks	72.4 ± 22.3 (n = 16)	72.6 ± 22.3 (n = 12)	72.5 ± 15.6 (n = 28)	60.3 ± 21.7 (n = 16)	51.7 ± 20.9 (n = 6)	58.0 ± 16.6 (n = 22)

c) Mann-Whitney p-values (one-sided) for tumor volumes listed in (**b**).

	**M**	**F**	**M&F**
	**WT vs. cKO**	**WT vs. cKO**	**WT vs. cKO**
**25 weeks**	0.2167	0.2437	0.4790
**40 weeks**	0.3935	0.2914	0.4398
**51 weeks**	0.2796	0.2130	0.1306

d) We used one-sided Mann-Whitney tests to compare tumor volumes (tumor volume, mean ± s.e) of males and females for each genotype, at each time point. For p38δ-cKO$^{\Delta K}$, mean tumor volume was significantly increased for females compared to males at week 25 (see bolded text).

Tumor Volume, Mean ± s.e.

	**WT**			**p38δ-cKO$^{\Delta K}$**		
	**M**	**F**	**p-value**	**M**	**F**	**p-value**
**25 weeks**	8.7 ± 2.4 (n = 15)	12.6 ± 4.6 (n = 4)	0.137	**6.5 ± 1.8 (n = 20)**	**62.2 ± 26.6 (n = 9)**	**0.003 (**)**
**40 weeks**	28.4 ± 7.4 (n = 16)	45.1 ± 17.7 (n = 12)	0.455	29.1 ± 11.7 (n = 14)	66.4 ± 34.4 (n = 7)	0.075
**51 weeks**	72.4 ± 22.3 (n = 16)	72.6 ± 22.3 (n = 12)	0.211	60.3 ± 21.7 (n = 16)	51.7 ± 20.9 (n = 6)	0.320

Supplementary Tables S3

Histopathological evaluation of WT and p38δ-cKO$^{\Delta K}$ skin tumors collected after 51 weeks of the DMBA/TPA chemical skin carcinogenesis regimen.

a) Incidence of malignant tumors: % (mice bearing malignant tumors) / (total mice)

Incidence	WT	p38δ-cKO$^{\Delta K}$
Mice with malignant tumors (SCC and/or KA)	84% (16/19)	47% * (9/19)

* cKO differs from WT at one-sided p < 0.05 by Fisher's exact test.

b) Tumor Multiplicity at 51 weeks, Mean ± s.e. Comparisons with significant p-values < 0.05 are highlighted in bold (p-values are shown in panel **(c)** below)

	Tumors per Animal Examined			
	WT		p38δ-cKO$^{\Delta K}$	
	M	F	M	F
**# examined**	10	9	8	11
**SCC**	0.70 ± 0.26	0.67 ± 0.17	0.38 ± 0.18	0.55 ± 0.21
**KA**	0.70 ± 0.26	**0.44 ± 0.18**	0.75 ± 0.53	**0.00 ± 0.00**
**PAP**	0.50 ± 0.27	0.33 ± 0.24	**1.00 ± 0.38**	**0.18 ± 0.12**
**Seb.Ad**	0.10 ± 0.10	0.11 ± 0.11	0.00 ± 0.00	0.00 ± 0.00
**Mel**	0.10 ± 0.10	0.33 ± 0.17	0.00 ± 0.00	0.00 ± 0.00
**Total**	2.10 ± 0.60	**1.89 ± 0.42**	2.12 ± 0.85	**0.73 ± 0.24**
**Malignant**	1.50 ± 0.40	**1.44 ± 0.29**	1.12 ± 0.61	**0.55 ± 0.21**
**Benign**	0.60 ± 0.34	0.44 ± 0.24	**1.00 ± 0.38**	**0.18 ± 0.12**
**# Tumor-free**	2	1	2	5

c) One-sided Mann-Whitney p-values for tumor multiplicities listed in **(b)**; significant p-values < 0.05 are highlighted in bold. SCC, squamous cell carcinoma; KA, keratoacanthoma; PAP, papilloma; Seb.Ad., Sebaceous adenoma; Mel, melanoma; Malign., malignant.

**Tumors per Animal Examined**		**SCC**	**KA**	**PAP**	**Seb. Ad.**	**Mel**	**Total**	**Mal-ign.**	**Ben-ign**
**Males**	**cKO vs. WT**	0.272	0.289	0.145	0.556	0.556	0.448	0.169	0.167
**Females**	**cKO vs. WT**	0.311	**0.026**	0.421	0.450	0.074	**0.019**	**0.017**	0.276
**cKO**	**Males vs. Females**	0.422	0.164	**0.035**	1.000	1.000	0.102	0.345	**0.035**
**WT**	**Males vs. Females**	0.459	0.322	0.444	0.737	0.249	0.527	0.461	0.500

Supplementary Table S4

Incidence of malignant tumors in WT and p38δ-cKO$^{\Delta M}$ males at 51 weeks post-DMBA.

% (mice bearing malignant tumors) / (total mice)

**Incidence**	**WT Males**	**p38δ-cKO$^{\Delta M}$ Males**
Mice with malignant tumors (SCC and/or KA)	78% (7/9)	33% (4/12) #

approaching significance at p = 0.0563 by Fisher's exact test (one-sided)

Supplementary Table S5

The expression of the genes linked to the Myeloid Leukocyte Activation gene category was significantly downregulated by p38δ ablation in v-ras^{Ha} transformed keratinocytes [1].

The following genes were downregulated:

Gene Symbol	Ratio (p38δ-KO vs Control)	Description
SLC11A1	-4.1	solute carrier family 11 (proton-coupled divalent metal ion transporters), member 1
CPLX2	-1.4	complexin 2
FCGR2B	-5.8	Fc receptor, IgG, low affinity IIb
SLC7A2	-1.8	solute carrier family 7 (cationic amino acid transporter, y+ system), member 2
TICAM1	-1.7	toll-like receptor adaptor molecule 1
SNCA	-3.1	synuclein, alpha
TGFBR2	-3.1	transforming growth factor, beta receptor II
MYO1F	-4.6	myosin IF (unconventional class I myosin 1f)
FCER1G	-2.1	Fc receptor, IgE, high affinity I, gamma polypeptide
IRF4	-1.9	interferon regulatory factor 4
FCGR3	-3.3	Fc receptor, IgG, low affinity III (CD16)

Reference:

1. Kiss, A.; Koppel, A.C.; Anders, J.; Cataisson, C.; Yuspa, S.H.; Blumenberg, M.; Efimova, T. Keratinocyte p38δ loss inhibits Ras-induced tumor formation, while systemic p38δ loss enhances skin inflammation in the early phase of chemical carcinogenesis in mouse skin. *Mol Carcinog* **2016**, *55*, 563–574.
